# Skin-interfaced microfluidic system with personalized sweating rate and sweat chloride analytics for sports science applications

**DOI:** 10.1126/sciadv.abe3929

**Published:** 2020-12-11

**Authors:** Lindsay B. Baker, Jeffrey B. Model, Kelly A. Barnes, Melissa L. Anderson, Stephen P. Lee, Khalil A. Lee, Shyretha D. Brown, Adam J. Reimel, Timothy J. Roberts, Ryan P. Nuccio, Justina L. Bonsignore, Corey T. Ungaro, James M. Carter, Weihua Li, Melissa S. Seib, Jonathan T. Reeder, Alexander J. Aranyosi, John A. Rogers, Roozbeh Ghaffari

**Affiliations:** 1 Gatorade Sports Science Institute, PepsiCo R&D Life Sciences, Barrington, IL 60010, USA.; 2 Epicore Biosystems Inc, Cambridge, MA 02139, USA.; 3 Querrey Simpson Institute for Bioelectronics, Northwestern University, Evanston, IL 60208, USA.; 4 Center for Bio-Integrated Electronics, Northwestern University, Evanston, IL 60208, USA.; 5 Gatorade Sports Science Institute, PepsiCo R&D Life Sciences, Bradenton, FL 34210, USA.; 6 Gatorade Sports Science Institute, PepsiCo R&D Life Sciences, Leicester, UK.; 7 Knight Campus for Accelerating Scientific Impact, 6231 University of Oregon, Eugene, OR 97403, USA.; 8 Department of Biomedical Engineering, Northwestern University, Evanston, IL 60208, USA.; 9 Departments of Mechanical Engineering, Electrical and Computer Engineering, and Chemistry, Northwestern University, Evanston, IL 60208, USA.; 10 Department of Neurological Surgery, Northwestern University Feinberg School of Medicine, Chicago, IL 60611, USA.; 11 Institute for Innovations in Developmental Sciences, Northwestern University, Chicago, IL 60611, USA.

## Abstract

Advanced capabilities in noninvasive, in situ monitoring of sweating rate and sweat electrolyte losses could enable real-time personalized fluid-electrolyte intake recommendations. Established sweat analysis techniques using absorbent patches require post-collection harvesting and benchtop analysis of sweat and are thus impractical for ambulatory use. Here, we introduce a skin-interfaced wearable microfluidic device and smartphone image processing platform that enable analysis of regional sweating rate and sweat chloride concentration ([Cl^−^]). Systematic studies (*n* = 312 athletes) establish significant correlations for regional sweating rate and sweat [Cl^−^] in a controlled environment and during competitive sports under varying environmental conditions. The regional sweating rate and sweat [Cl^−^] results serve as inputs to algorithms implemented on a smartphone software application that predicts whole-body sweating rate and sweat [Cl^−^]. This low-cost wearable sensing approach could improve the accessibility of physiological insights available to sports scientists, practitioners, and athletes to inform hydration strategies in real-world ambulatory settings.

## Teaser

Wearable microfluidic system enables remote analysis of sweat rate and chloride loss for assessing personalized fluid needs.

## INTRODUCTION

Advances in materials science, mechanics design, and miniaturized electronics serve as the foundations for emerging classes of thin, soft skin-interfaced devices for multifunctional sensing of physiological status and processes (*1*, *2*). Biochemical analysis of sweat in situ represents a promising pathway for enabling intermittent and continuous monitoring of sweat loss and composition, which is important for maintaining proper hydration and electrolyte balance, particularly in athletic contexts (*3*). Precise, real-time measurements of sweat dynamics (i.e., local sweating rate and local total sweat volume) and sweat biomarkers require wearable chemical systems capable of continuous capture and analysis of sweat and transmission of the resulting information locally to the user or remotely to health professionals (*4*, *5*). A critical requirement for the broad adoption of such wearable systems is in the ability to reliably collect and measure analytes with minimal contamination. Conventional technologies for sweat collection have relied on absorbent pads, gauzes, and centrifuge systems, with the need for external laboratory instruments for analysis. These approaches support basic performance and physiology studies within controlled laboratory settings, but they are not suitable for real-time and ambulatory deployments.

The specific focus of the current work is on human performance and athletics, where body fluid and electrolyte deficits accrued through sweat loss during physical activity and heat stress increase cardiovascular strain, which, in turn, could lead to impairment of physical and cognitive performance (*6*–*10*). Because of the considerable variation in sweating rate (~0.5 to 3 liters/hour) and sweat electrolyte concentrations [sodium ([Na^+^]) and chloride ([Cl^−^]) ~10 to 100 mM] (*11*, *12*), personalized fluid replacement strategies based on individual sweat profiles are recommended (*9*, *13*). Whole-body sweat loss is typically estimated via the measurement of change in body mass before and after exercise while also accounting for any fluid intake and/or urine loss during the test session. The reference technique for whole-body measurement of sweat electrolyte concentrations is the washdown procedure (*14*–*16*). Such approaches are lengthy, retrospective, and require athlete and practitioner adherence to tight quality control procedures. Thus, there has been recent interest in regional techniques to estimate whole-body sweating rate and electrolyte loss. Still, assessing sweat profiles using established regional sweat collection and analysis techniques is a slow, labor-intensive process and impractical for ambulatory use. For example, hygrometry is considered the gold standard technique for measuring regional sweating rate, but ventilated sweat capsules require specialized, wired equipment, and controlled laboratory conditions (*16*). Furthermore, established gravimetric-based techniques such as filter paper, sweat pouches, and plastic sweat collectors are not conducive to real-world applications such as on-field sports training (*16*). While the absorbent patch technique has been widely used with athletes to measure sweat electrolyte concentrations (*11*, *17*–*19*), the required post-collection harvesting and expensive benchtop analysis of sweat is impractical for the general population and precludes real-time feedback to the wearer.

The accurate measurement of sweat dynamics requires the effective isolation of sweat from the skin and the surrounding environment to seal the sweat from contaminants. Skin-like, lab-on-a-chip microfluidic platforms are, therefore, of particular interest, because of their ability to collect, route, and chemically analyze precise, microliter volumetric samples of sweat released from well-defined regions of the skin. The integration of microfluidics directly with the surface of the skin supports many important operations in fluidic manipulation of sweat for precise capture, storage, volumetric measurement, and chemical analysis. The development of wearable systems that can monitor biomarkers in situ via electrochemical sensors represents a promising pathway for continuous monitoring of sweat, whereby sweat is routed to sensing electrodes that interface to recording electronics, power supply systems, and radio communication hardware (*4*, *20*–*24*). Colorimetric biochemical sensors provide a unique set of advantages in this context including methods of multianalyte analysis (*5*), hybrid operation (*23*), and time-correlated sampling (*25*, *26*) all in low-cost (*27*) and waterproof form factors (*28*). A key feature of colorimetric sensing is a readout mode that provides data directly to the user or study administrator via the naked eye or quantitatively via image capture with a smartphone after calibrating for environmental lighting conditions (*29*). A set of additional technical requirements for wearable sweat sensors involves the use of physical designs, which align with conventional manufacturing workflows. Roll-to-roll manufacture is one option (*30*) and has been used in a pilot study for sweat monitoring of 40 subjects (*20*). However, the utility of wearable sweat sensors to track sweating rate and sweat electrolyte loss has yet to be demonstrated in a large, diverse cohort in uncontrolled environments.

The primary objective of this work was to determine the clinical validity of a roll-to-roll manufacturable, skin-interfaced wearable microfluidic device with colorimetric sensors and a smartphone image processing platform in measuring regional sweating rate and sweat [Cl^−^]. The absorbent sweat patch technique was used as the reference method since it has been established as a reliable measure of regional sweating rate and sweat [Cl^−^] and is the well-accepted method for individualized sweat electrolyte testing in the field (*16*). Another objective of this study was to develop algorithms to predict whole-body sweating rate and whole-body sweat [Cl^−^] in a large clinical study (312 athletes), which is an important step in using skin-interfaced wearable microfluidic devices to help determine individualized fluid-electrolyte replacement needs.

## RESULTS

### Soft wearable microfluidic patch design and sensing strategies

**Fig. 1:**
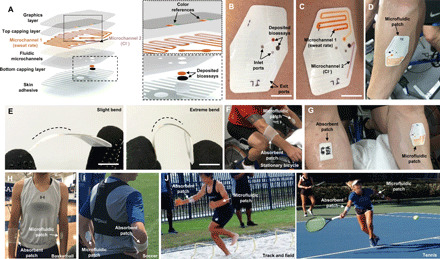
Schematic drawings, optical images, and field studies of wearable microfluidic patch. (**A**) Exploded view illustration of microfluidic patch and its subassembly layers. (Insets) Magnified images of the reference colors in the top graphics layer (top) and deposited assays in the embedded layer (bottom). (**B**) Optical image of microfluidic patch on the ventral forearm before exercise (unfilled) (scale bar, 1 cm). (**C**) Optical image of microfluidic patch showing sweat filling in microchannels 1 and 2 (scale bar, 1 cm). (**D**) Optical image of microfluidic patch (zoom-out view) showing the device filling as sweat is excreted on the forearm. (**E**) Optical images of microfluidic patch under slight bending (left) and extreme bending (right) (scale bar, 1 cm). (**F**) Optical image of a cyclist wearing a microfluidic patch and an absorbent patch on opposing arms during exercise on a stationary bicycle in controlled laboratory environment. (**G**) Close-up image of a microfluidic patch and an absorbent patch taken during exercise. (**H** to **K**) Optical images of subjects wearing a microfluidic patch and an absorbent patch during different sports (basketball, soccer, track and field, and tennis) under uncontrolled environmental conditions. Photo credit: Stephen Lee, Epicore Biosystems.

The wearable microfluidic patch technology introduced here involves multilayered stacks of thin-film polymers that contain intricate microfluidic channels created using laser and die cutting techniques. The network of microchannels and assay wells are created using roll-to-roll processing of polymeric rolls of materials, allowing for rapid (~1000 patches/min) and low-cost manufacturing of soft conformal microfluidic constructs, as an alternative to silicone-based mold casting techniques. The microfluidic channels are composed of hydrophobic polymeric materials that route sweat by exploiting the natural pressure associated with eccrine sweat excretion. Figure 1A shows the multilayered microfluidics, dye and bioassay reservoirs, the top graphics layer with color reference stripes, and a subjacent skin adhesive layer, which collectively define the low-modulus features of the flexible sticker-like patch. Microchannel 1 has the capacity to collect ~130 μl of sweat from a defined sweat collection region (38.5 mm^2^ and 7 mm diameter). An orange dye mixes with sweat to make propagation along the channel highly visible, allowing rapid assessment and measurement of sweat volume (Fig. 1A, inset). In contrast, microchannel 2 has a smaller capacity (~30 μl) and collection area (12.6 mm^2^ and 4 mm diameter) designed to support a colorimetric reaction between excreted sweat entering the microchannel and deposited chemical reagents for analysis of [Cl^−^]. Figure 1B shows a representative example of the microfluidic patch (without the top graphics layer) skin-mounted on the ventral forearm before exercise begins. During exercise, microchannels 1 and 2 capture and mix sweat as shown in Fig. 1C. The spatial extent of orange sweat capture in microchannel 1 and the purple color intensity in microchannel 2 provide a measure of local sweat excretion volume and sweat [Cl^−^], respectively. Figure 1D shows an optical image of the microfluidic patch on another subject with defined vein contours on the ventral forearm. The microfluidic patch intimately conforms to the surface of the skin without causing irritation around curvilinear regions or in the presence of heavy sweat excretion. The thin geometry (~680 μm) and low bending stiffness of the device support mechanical deformations (Fig. 1E), aiding wearability during intense physical activities.

The colorimetric sensing strategy used in the microfluidic patch provides quantitative assessment of regional sweat loss, sweating rate, and sweat [Cl^−^] in real-world settings. To characterize the accuracy of this sensing approach in demanding and intense exercise scenarios, the microfluidic patch performance was assessed in comparison to conventional sweat analysis techniques using absorbent patches for collection of sweat and subsequent benchtop gravimetry (for sweat volume) and ion chromatography (for sweat [Cl^−^]) techniques. Figure 1F shows a subject exercising on a cycle ergometer while instrumented with a microfluidic patch (left forearm) and absorbent patch (right forearm). The magnified image of the forearms in Fig. 1G highlights the visual nature of the microfluidic patch compared to the absorbent patch. In addition to controlled activities in a laboratory, microfluidic patch performance was compared with absorbent patches in uncontrolled environments and across different physically demanding sports, including lacrosse, basketball (Fig. 1H), soccer (Fig. 1I), track and field (Fig. 1J), and tennis (Fig. 1K).

### Smartphone-based image processing and real-time colorimetric analysis

**Fig. 2:**
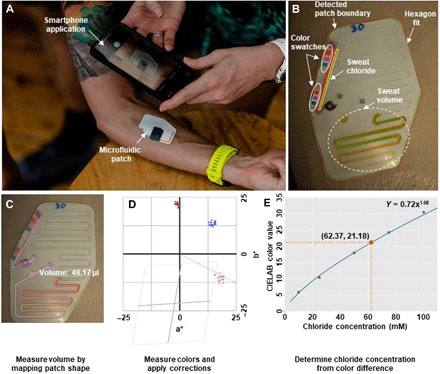
Automated optical analysis of microfluidic patch. (**A**) Person photographing the patch on user’s arm with the smartphone application. (**B**) Automated detection of patch boundaries and critical features. Colored outlines denote boundaries of detected patch features. (**C**) Image is mapped to known patch shape for volume measurement. (**D**) Measured CIELAB colors for the chloride channel (blue), nearby patch background (red), and difference after color correction (purple). The purple line shows the vector of the expected color difference. The gray lines show the effect of color correction on the center four grid boxes. (**E**) Color vector length maps monotonically to chloride concentration (average vector length from Fig. 2D is denoted in red). Photo credit: Alexander J. Aranyosi, Epicore Biosystems.

Digital image capture and analysis with a smartphone enable simple and rapid assessment of instantaneous sweating rate and sweat [Cl^−^] from the microfluidic patch in ambulatory settings. Custom software uses the smartphone camera to capture and analyze the microfluidic patch as shown in Fig. 2A. This technique enables detection of the microfluidic patch boundary in the image frame, as well as the positions of the microchannels and reference color markers (Fig. 2B). The reference color markers allow ambient light correction and white balancing in real time, thereby eliminating the effects of variable lighting conditions (e.g., daylight, shadows, and cloudy environments). Upon recognition of the microfluidic patch landmarks, the software application detects the orange color in microchannel 1 and computes collected sweat volume based on the spatial distribution of the orange color and the three-dimensional patch geometry (Fig. 2C). Following correction of the measured colors using the reference markers, the intensity of the purple color in microchannel 2 is measured in CIELAB color space relative to the patch background across multiple regions of interest (Fig. 2, D and E). Light absorption varies monotonically with the concentration of the colorimetric assay according to the Beer-Lambert law. Since this concentration, in turn, is proportional to [Cl^−^] of collected sweat, [Cl^−^] can be estimated from the color intensity. This technique measures local sweat volume and sweat [Cl^−^] with a resolution of 0.01 μL and 0.1 mM, respectively.

### Microfluidic patch versus absorbent patch during on-field/court sports training

**Fig. 3:**
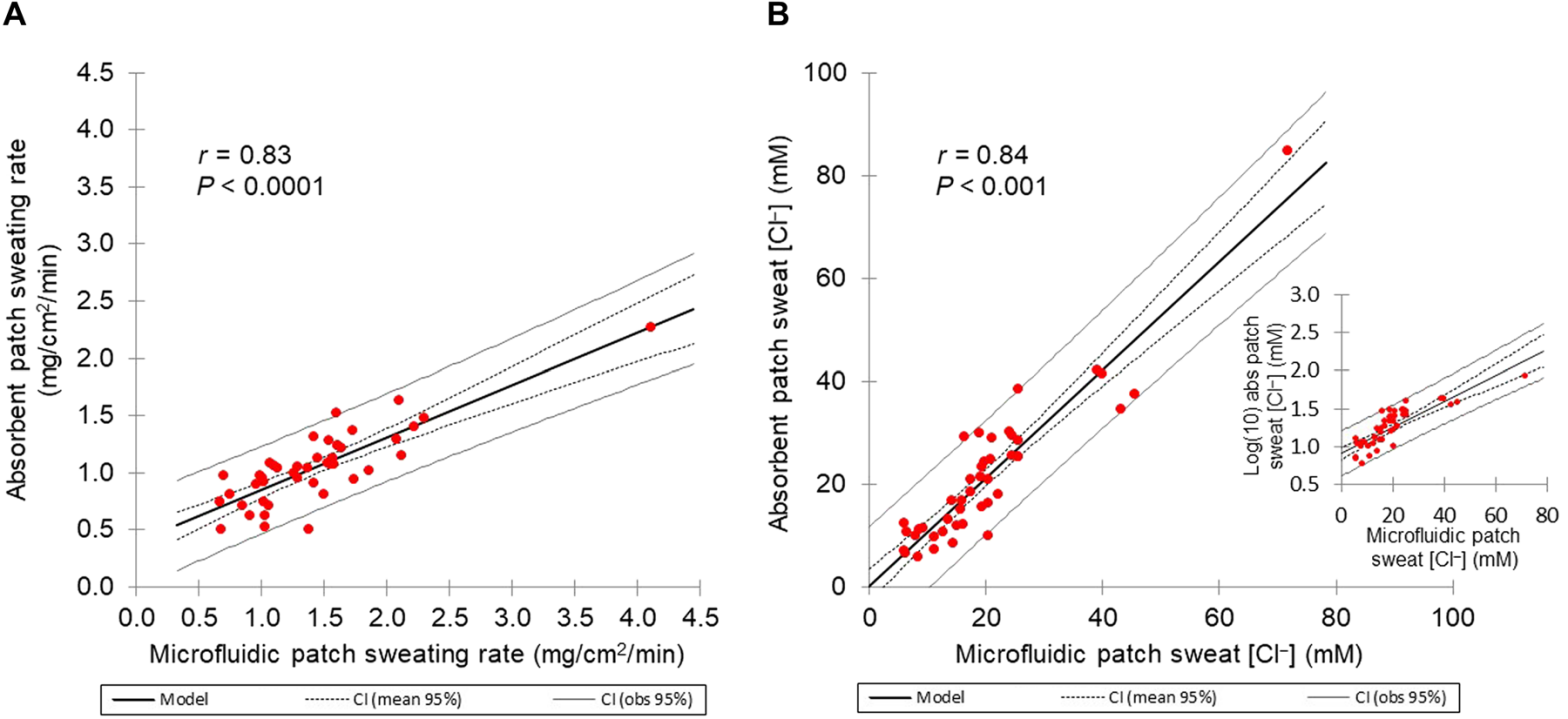
Scatterplots of the microfluidic versus absorbent patch data with competitive athletes during on-field/court sports training. (**A**) Regional sweating rate measurements and (**B**) regional sweat chloride concentration under varying environmental and ambient lighting conditions (*n* = 43 subjects). Assumptions for homogeneity of variance of the absorbent patch sweat chloride concentration were not met (B). Therefore, the inset of (B) shows the scatterplot and correlation analysis results of raw microfluidic sweat chloride concentration versus log-transformed absorbent patch sweat chloride concentration. Note that when the outlier data point with a high regional sweating rate and high regional sweat [Cl^−^] is removed from (A) and (B), the Pearson correlations remain statistically significant (*r* = 0.73 and *r* = 0.82, respectively; *P* < 0.001).

To test the accuracy of the microfluidic patch and the accompanying software, the microfluidic patch and absorbent patches were used to measure regional sweating rate (Fig. 3A) and sweat [Cl^−^] (Fig. 3B) from competitive athletes during on-field/court sports training under varied environmental and ambient lighting conditions (*n* = 43 subjects). Microfluidic patch results were significantly correlated with absorbent patch data for both regional sweating rate (*r* = 0.83, *P* < 0.0001) and sweat [Cl^−^] (*r* = 0.84, *P* < 0.001). When the outlier data point with a high regional sweating rate and high regional sweat [Cl^−^] is removed from Fig. 3 (A and B), the Pearson correlations remain statistically significant (*r* = 0.73 and *r* = 0.82, respectively; *P* < 0.001). While the microfluidic patch sweating rate was higher than that of the absorbent patch (1.42 ± 0.60 versus 1.04 ± 0.33 mg/cm^2^ per minute, *P* < 0.0001), there was no difference in sweat [Cl^−^] between the microfluidic and absorbent patches (21.4 ± 14.1 versus 20.0 ± 12.4 mM, *P* = 0.11). The strong correlation between the microfluidic and absorbent patches, which represent two different methodologies, demonstrates the robustness of the microfluidic patch across a diverse group of athletes.

There were originally 55 participants in this study. Data from one subject were excluded because the absorbent patch was on the skin too long and became oversaturated. Eleven subjects’ data (20%) were excluded from analysis because of the following microfluidic device failures: (i) Sweat did not advance far enough in microchannel 2 by the end of exercise (*n* = 5), (ii) the patch delaminated or fell off (*n* = 4), or (iii) clouding or backflow in microchannel 1 occurred likely from physical impact to the patch during training (*n* = 2). Therefore, the final dataset was *n* = 43 (fig. S1).

### Regional microfluidic patch and whole-body sweating rate and chloride measurements

**Fig. 4:**
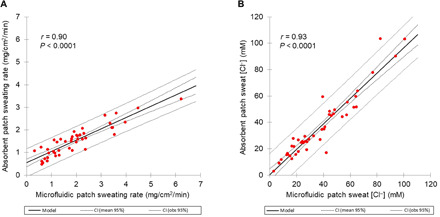
Scatterplots of the microfluidic versus absorbent patch data under controlled laboratory conditions. (**A**) Regional sweating rate data and (**B**) regional sweat chloride concentration measured during cycling on a stationary bicycle (*n* = 45 subjects).

To examine the relation between regional and whole-body sweat, subjects wore microfluidic patches and absorbent patches in a controlled laboratory environment where whole-body sweat measurements were also collected. Microfluidic patch results (*n* = 45 subjects) were significantly correlated with the absorbent patch for both regional sweating rate (*r* = 0.90, *P* < 0.0001; Fig. 4A) and sweat [Cl^−^] (*r* = 0.93, *P* < 0.0001; Fig. 4B). Microfluidic patch sweating rate was significantly higher than that of the absorbent patch (1.99 ± 1.22 versus 1.55 ± 0.68 mg/cm^2^ per minute, *P* < 0.0001), as was observed in on-field/court sports (Fig. 3). There was no difference in sweat [Cl^−^] between the microfluidic and absorbent patches (37.8 ± 23.3 versus 36.7 ± 23.7 mM, *P* = 0.32).

**Fig. 5:**
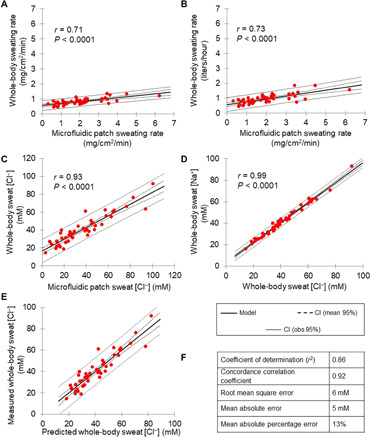
Scatterplots of microfluidic regional sweat versus whole-body sweat under controlled laboratory conditions (*n* = 45 subjects). (**A**) Microfluidic regional versus whole-body sweating rate, expressed relative to body surface area (milligrams per square centimeter per minute). (**B**) Microfluidic regional versus whole-body sweating rate, with whole-body sweating rate expressed in absolute terms (liters per hour). (**C**) Microfluidic regional sweat chloride concentration versus whole-body sweat chloride concentration. (**D**) Whole-body sweat chloride concentration versus whole-body sweat sodium concentration. (**E**) Whole-body sweat chloride concentration model showing predicted versus measured whole-body sweat chloride concentration. (**F**) Metrics for the model predicting whole-body sweat chloride concentration from microfluidic sweat chloride concentration. Note that when the outlier data point with a high microfluidic patch sweating rate is removed from (A) and (B), the Pearson correlation results remain statistically significant (*r* = 0.70 and *r* = 0.71, respectively; *P* < 0.0001).

Figure 5 (A and B) shows scatterplots of microfluidic regional sweating rate versus whole-body sweating rate under controlled laboratory conditions (*n* = 45 subjects). Whole-body sweating rate data are expressed in body surface area–normalized (milligrams per square centimeter per minute, *r* = 0.71, *P* < 0.0001; Fig. 5A) and absolute (liters per hour, *r* = 0.73, *P* < 0.0001; Fig. 5B) values. Means ± SD body surface area–normalized whole-body sweating rate was 0.82 ± 0.22 mg/cm^2^ per minute, and absolute sweating rate was 0.95 ± 0.32 liters/hour. When the outlier data point with a high microfluidic patch sweating rate is removed from Fig. 5 (A and B), the Pearson correlation results remain statistically significant (*r* = 0.70 and *r* = 0.71, respectively; *P* < 0.0001).

The results shown in Fig. 5 (A and B) suggest that the correlation between microfluidic and whole-body sweating rate is similar regardless of whether or not the data are normalized to body surface area. Therefore, all whole-body sweating rate models hereafter are focused on the prediction of sweating rate in absolute terms (liters per hour) for ease of practical interpretation.

Figure 5C includes a scatterplot of microfluidic regional sweat [Cl^−^] versus whole-body sweat [Cl^−^] under controlled laboratory conditions (*n* = 45 subjects: *r* = 0.93, *P* < 0.0001). A scatterplot of whole-body sweat [Cl^−^] versus whole-body sweat [Na^+^] under controlled laboratory conditions (*n* = 45 subjects: *r* = 0.99, *P* < 0.0001) is shown in Fig. 5D. Whole-body sweat [Cl^−^] was 41.3 ± 16.5 mM (means ± SD), and whole-body sweat [Na^+^] was 41.8 ± 15.5 mM. Establishing the relation between whole-body sweat [Cl^−^] and [Na^+^] is relevant because published recommendations for electrolyte replacement are based on sweat Na^+^ losses (*9*, *13*). The results suggest that there is a strong relation between whole-body sweat [Cl^−^] and [Na^+^] [*r*^2^ = 0.98, concordance correlation coefficient (CCC) = 0.99, mean absolute agreement (MAE) = 2 mM, and root mean square error (RMSE) = 2 mM]. Figure 5E shows predicted versus measured whole-body sweat [Cl^−^] using the model established via the simple linear regression analysis (depicted in Fig. 5C). Prediction model metrics are also shown in Fig. 5F for whole-body sweat [Cl^−^] (*r*^2^ = 0.86, CCC = 0.92, MAE = 5 mM, and RMSE = 6 mM).

In this study, there were originally 49 participants. Data from 4 subjects (8%) were excluded from analysis because sweat did not advance far enough in the microfluidic patch channel 2 by the end of exercise (*n* = 3) or the microfluidic patch delaminated (*n* = 1). Therefore, the final dataset was *n* = 45 (fig. S2).

### Day-to-day coefficient of variation of the microfluidic patch in measuring regional sweating rate and sweat chloride concentration

Reliability of the microfluidic and absorbent patch methods for measuring regional sweating rate and sweat [Cl^−^] under controlled laboratory conditions (*n* = 12) are shown in Table 1. Coefficients of variation (CVs) were similar between methods for sweating rate (CV = 9% for both methods) and sweat [Cl^−^] (microfluidic CV = 12% and absorbent patch CV = 13%). Whole-body sweating rate was not different between days (1.07 ± 0.50 and 1.09 ± 0.49 liters/hour, *P* = 0.47), and the CV was 4%.

**Table 1:** Day-to-day variation in sweating rate and sweat chloride concentration when measured via the absorbent patch and microfluidic patch.

	**Absorbent patch** **sweating rate**	**Microfluidic patch** **sweating rate**	**Absorbent patch** **sweat [Cl^−^]**	**Microfluidic patch** **sweat [Cl^−^]**
Day 1 (means ± SD)	1.21 ± 0.40 mg/cm^2^/min	1.96 ± 0.96 mg/cm^2^/min	30.2 ± 12.5 mM	37.6 ± 14.7 mM
Day 2 (means ± SD)	1.21 ± 0.31 mg/cm^2^/min	2.13 ± 1.00 mg/cm^2^/min	31.4 ± 13.9 mM	36.4 ± 14.3 mM
Difference between days 1 and 2 (means ± SD)	0.00 ± 0.19 mg/cm^2^/min	0.17 ± 0.30 mg/cm^2^/min	1.2 ± 6.0 mM	−1.2 ± 8.5 mM
*P* value	0.73	0.08	0.50	0.60
CV	8.8%	8.9%	12.8%	11.6%

### Development of a whole-body sweating rate predictive model based on regional measurements

**Fig. 6:**
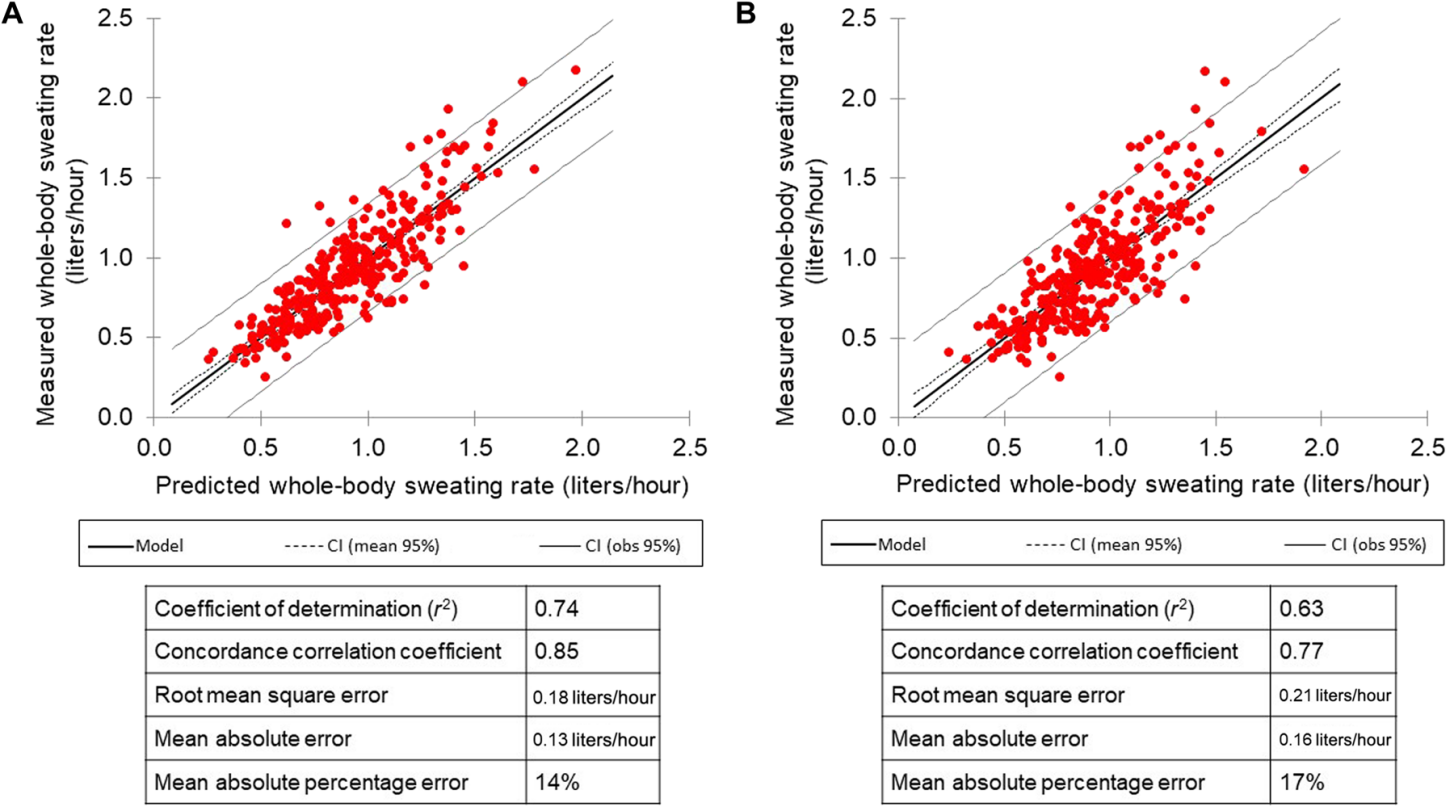
A scatterplot showing predicted versus actual whole-body sweating rate data for recreational and competitive athletes tested under a range of environmental conditions. (**A**) Whole-body sweating rate results with a seven-factor model including microfluidic regional sweating rate, body mass, sex, air temperature, type of sport, exercise duration, and energy expenditure (*n* = 312 subjects) across various team and individual sports. (**B**) Whole-body sweating rate results with a six-factor model including all of the above except energy expenditure (*n* = 312 subjects).

A model was derived to predict whole-body sweating rate from microfluidic regional sweating rate data in recreational to competitive athletes (*n* = 312) of various team and individual sports tested under a range of environmental conditions (Fig. 6). Inputs to the model included microfluidic regional sweating rate and various factors related to subject characteristics (body mass and sex), environment (air temperature), and exercise conditions (type of sport, energy expenditure, and exercise duration). Figure 6A shows results with the model that includes all seven input factors (*r*^2^ = 0.74, CCC = 0.85, MAE = 0.13 liters/hour, and RMSE = 0.18 liters/hour). Figure 6B shows results for a six-factor model (all inputs except energy expenditure) (*r*^2^ = 0.63, CCC = 0.77, MAE = 0.16 liters/hour, and RMSE = 0.21 liters/hour). In this study, means ± SD microfluidic sweating rate was 1.25 ± 0.79 mg/cm^2^ per minute, and whole-body sweating rate was 0.92 ± 0.33 liters/hour.

There were originally 346 participants in this study. Eleven subjects’ data were excluded because of equipment issues in obtaining energy expenditure in the field. Data from 23 subjects (7%) were excluded from analysis because of the following microfluidic patch failures: (i) Sweat did not advance far enough in microchannel 2 by the end of exercise (*n* = 9), (ii) the patch delaminated or fell off (*n* = 10), or (iii) backflow in microchannel 1 likely from physical impact to the patch during training (*n* = 4). Therefore, the final dataset was *n* = 312 (fig. S3).

## DISCUSSION

Systematic studies were conducted to compare the wearable microfluidic platform with standard techniques for sweat testing. The main finding was that regional sweating rate and sweat [Cl^−^] data from the microfluidic patch were significantly correlated with those of the standard absorbent patch technique during ~90 min of exercise under varying environmental and ambient lighting conditions. Furthermore, we investigated the test-retest (day-to-day) reliability of the microfluidic device, which is a requisite step in any methodological validation process. The CVs for the microfluidic device were similar to that of the reference techniques for both sweating rate (9%) and sweat [Cl^−^] (12 to 13%) in the present study. These CVs were also consistent with previous research investigating day-to-day variability in forearm sweating rate and electrolyte concentrations (*12*, *16*). This work improves upon previous feasibility studies with similar devices (*5*) and advances the field by demonstrating validation in hundreds of athletes (*n* = 312), not only in a controlled setting but also in competitive athletes during live on-field/court training for several sports.

Actionable hydration feedback from the microfluidic patch requires estimating whole-body sweating rate and sweat [Cl^−^] from the regional measurements. To this end, robust models based on microfluidic patch results and other available information were developed for implementation into a smartphone application. The good agreement between predicted and measured whole-body sweating rate (*r*^2^ = 0.74, CCC = 0.85) and sweat [Cl^−^] (*r*^2^ = 0.86, CCC = 0.92) provides additional validation of microfluidic patch measurements and enables personalized fluid-electrolyte intake recommendations for athletes (*12*). Results suggest that the mean absolute error of the prediction models are 0.13 liters/hour (or 14%) for whole-body sweating rate and 5 mmol (or 13%) for whole-body sweat [Cl^−^].

**Fig. 7:**
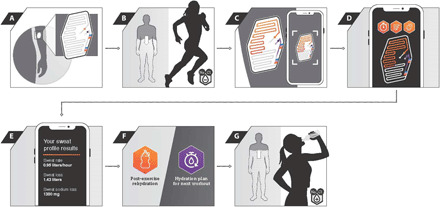
Schematic flow diagram of operation of the wearable microfluidic platform and smartphone application to determine personalized hydration recommendations. (**A**) The user applies the sweat patch on their left ventral forearm after cleaning and drying the skin surface. (**B**) Passive sweat collection and reaction with the colorimetric assays as the athlete completes their workout. (**C**) After exercise, the user takes an image of the patch via the smartphone application. (**D**) The application processes the patch results, pulls in other inputs (body mass and sex from the user’s profile, type of sport, exercise duration, weather data, and energy expenditure), and applies algorithms. (**E**) Sweat profile results, including whole-body sweating rate, whole-body sweat loss, and whole-body sweat sodium loss, are displayed on the screen (example shown is for a 90-min session). (**F**) Personalized fluid intake recommendations are provided on the basis of the user’s sweat profile. (**G**) User follows recommendations to properly rehydrate immediately after workout and properly hydrate during their next workout of similar intensity, duration, and environment.

Figure 7 presents a schematic flow of the system operation that uses the wearable microfluidic platform in combination with a smartphone application to determine sweat profile results and personalized hydration recommendations. This system improves on time-intensive and laborious conventional sweat analysis methods and consists of (i) placement of a soft microfluidic patch on an easily accessible area of the body (left ventral forearm), (ii) passive sweat collection and reaction with colorimetric assays, (iii) image capture of colorimetric responses with a smartphone, (iv) image analysis via computer vision and application of predictive algorithms, (v) generation of sweat profiles, and (vi) development of personalized hydration strategies for (vii) optimizing post-workout rehydration and fluid intake during future exercise sessions.

To standardize testing across the large population of subjects and multiple trials, we used a set of best practices developed for the microfluidic patch that were applicable to both controlled laboratory and uncontrolled environments. For adequate adhesion of the microfluidic patch to the skin, it is critical that the skin is clean, free of skin-care products (lotions, sunscreen, etc.), and dry before device application. In addition, while we successfully tested athletes in the field (trials 1 and 3) without having to shave the patch site, it is prudent for individuals with high hair follicle density on their ventral forearm to shave the area before patch application. While wearing the patch, the user is instructed to avoid physically probing the microfluidic channels (e.g., from towel drying the skin) or peeling the patch from the skin (e.g., contact sports), which could lead to device failures.

Future research is needed to corroborate the validity of the microfluidic sweat patch and broaden its utility even further. As noted above, the microfluidic patch could not measure sweat [Cl^−^] in a small percentage of subjects (<10%) because sweat did not advance far enough in microchannel 2 to initiate the colorimetric reaction. Research is planned to enlarge the collection area of future versions of the microfluidic patch to accommodate low sweat flow rates (≤0.4 mg/cm^2^ per minute). In addition, while the exercise duration in the present studies (up to ~1.5 to 2 hours) was representative of typical workouts by recreational and trained athletes (e.g., running for fitness or training, team sport practice for soccer, basketball, etc.), the results may not be applicable to endurance events lasting longer than 1.5 to 2 hours. Therefore, future research with a focus on longer duration testing is needed to confirm the validity of the microfluidic patch during exercise that extends beyond 1.5 to 2 hours. Other potential avenues of future research with this device include validation and algorithm development for patch application to the right forearm and other regions of the body and for a broader range of environmental conditions and additional types of sports/physical activities.

In conclusion, the microfluidic patch enables real-time assessment of sweating rate and sweat [Cl^−^] under field conditions with no need for specialized expertise or laboratory tools. Collection of sweating rate and sweat electrolyte loss data using this low-cost wearable sensing approach could improve the accessibility of physiological insights available to sports scientists, practitioners, and athletes to inform hydration strategies in real-world settings, with applications not only in athletic performance and fitness but also in military readiness and clinical medicine.

## MATERIALS AND METHODS

### Study design

A series of trials was carried out to compare the wearable microfluidic platform with standard techniques for sweat testing. The objective of trial 1 was to compare the microfluidic patch and absorbent patch results for regional sweating rate and sweat [Cl^−^] during on-field/court sports training. In trial 2a, the objectives were (i) to compare the microfluidic patch and absorbent patch results for regional sweating rate and sweat [Cl^−^] and (ii) to determine the relation between the microfluidic patch and whole-body sweating rate and sweat [Cl^−^]. The objective of trial 2b was to determine the day-to-day CV of the microfluidic patch in measuring regional sweating rate and sweat [Cl^−^]. Last, in trial 3, the objective was to develop a whole-body sweating rate predictive model.

This research (clinical trial identifier: NCT04240951) was approved by the Sterling Institutional Review Board (IRB) (Atlanta, GA) for the protection of human study participants (Sterling IRB ID: 6004). Each participant and his/her parent or guardian (for subjects under 18 years) were informed of the experimental procedures and associated risks before providing written informed consent. Figures S1 to S3 show the CONSORT flow diagrams for study enrollment, participant exclusion, and data exclusion details for trials 1, 2, and 3, respectively. In total, data from 312 subjects (194 males and 118 females; 15 to 45 years) were analyzed. Participants ranged from recreationally active to highly trained athletes competing in individual or team sports. Table S1 provides summary data of subject characteristics for each trial.

### Fabrication of wearable microfluidic devices

Standard laser and die cutting techniques, which support roll-to-roll manufacturing processes, enabled fabrication of wearable microfluidic devices. Briefly, a five-layer stack of thin-film elastic polymers formed the microfluidic channels, the top graphics layer, and patterned skin adhesive layer. Pressure-sensitive adhesive served as the intermediate to bond the individual layers together. Inlet windows and windows in the adhesive layer created openings to define the two sweat collection regions interfacing with the skin. For colorimetric analysis, a dehydrated colored dye (~4 μl) was deposited in microchannel 1 to measure regional sweat volume and sweating rate. The dye dissolves into sweat as it passes, creating an orange streak whose front can be measured to determine collected sweat volume. Microchannel 2 was prepared with a Cl^−^-sensitive bioassay consisting of a 5-μl volume of a mixture of silver chloranilate and polyhydroxyethylmethacrylate (pHEMA; Sigma-Aldrich, MO, USA) in methanol (2%, w/v) placed near the inlet region of the microchannel for Cl^−^ detection. The pHEMA creates a hydrogel that stabilizes the insoluble silver chloranilate. As sweat passes through the hydrogel, the Cl^−^ reacts with silver chloranilate to produce silver chloride, which precipitates out, and soluble chloranilic acid is carried with the sweat and produces a purple color with a concentration-dependent intensity.

### Smartphone and DSLR camera–based colorimetric analysis of sweating rate and chloride concentration

Photos of microfluidic patches were captured using a smartphone (iPhone 8, Apple Inc.) and digital single-lens reflex (DSLR) camera (EOS 6D, Canon Inc.). Images were captured at the time of absorbent patch removal, at the end of exercise, and wherever possible at earlier times during exercise. RAW images were used for processing to eliminate artifacts introduced by normalization, compression, and other preprocessing steps. Traditional computer vision algorithms identified the locations of relevant features including the patch outline, color swatches, and filled regions of microchannel 1. Features on the graphics layer allowed patch orientation to be determined, after which the patch outline and filled regions of microchannel 1 were fed into a computational model of the patch to determine filled volume. Normalized sweating rate was computed from this volume, the collection area defined by the patch adhesive, and the elapsed time from exercise start to photo capture. The color swatches were used to derive a mapping from the image color space to a known color space for Cl^−^ measurement. The colors of regions along microchannel 2 and corresponding background regions alongside were measured and mapped to this known color space. The intensity of purple within microchannel 2 relative to the patch background was then used to determine [Cl^−^]. These methods were validated by comparing them to measurements performed manually from the same images. Additional processing details are described in fig. S4.

### Regional sweating rate and sweat chloride concentration measurements

Each trial applied the following methods for measuring regional sweating rate (trials 1, 2, and 3) and regional sweat [Cl^−^] (trials 1 and 2). Sweat was collected from the right and left ventral forearms with an absorbent patch (Tegaderm+Pad, 3M, St. Paul, MN; pad size, 11.9 cm^2^) and the wearable microfluidic patch, respectively. This was deemed a fair comparison between methods since several previous studies have reported no significant bilateral differences in forearm sweating rate and sweat electrolyte concentrations (*12*, *31*–*34*). The CV between the left and right ventral forearms is ~12 to 13%, while the day-to-day CV in ventral forearm sweating rate and sweat [Cl^−^] is ~9 to 13% (*12*).

Before patch application, the ventral forearms were rinsed with deionized water and wiped dry with electrolyte-free gauze (10 × 10 cm, Thermo Fisher Scientific, Waltham, MA). For optimal patch adhesion to the skin in the laboratory study (trial 2), the ventral forearms were also shaved if needed to remove hair (~20% of subjects). For ecological validity, the ventral forearms were not shaved in the field testing. However, during field testing, an elastic net dressing (Surgilast, Derma Sciences, Princeton, NJ) was put on the right forearm to ensure that the absorbent patch remained adhered to the skin. Absorbent patches were removed upon moderate sweat absorption but before saturation as determined by visual inspection (patch time on skin was 39 to 112 min for trial 1 and 12 to 71 min for trial 2). Upon removal, the absorbent pad was immediately separated from the Tegaderm using clean forceps and placed in an air-tight plastic tube (Sarstedt Salivette). Regional sweating rate (in milligrams per square centimeter per minute) was measured gravimetrically on the basis of the mass of sweat absorbed in the pad (to the nearest 0.001 g using an analytical balance; Mettler Toledo Balance XS204, Columbus, OH), the pad surface area, and the duration that the patch was on the skin. Sweat from the absorbent patch was extracted via centrifuge and subsequently analyzed for [Cl^−^] in duplicate by ion chromatography (Dionex ICS-3000).

### Whole-body sweating rate and sweat electrolyte concentrations

For trials 1, 2, and 3, whole-body sweating rate was calculated from the difference in pre- to post-exercise body mass, corrected for food/fluid intake, urine/stool loss, respiratory water loss, and weight loss due to substrate oxidation (*35*), divided by exercise duration. Whole-body sweat [Na^+^] and [Cl^−^] were measured in trial 2a, and details of this methodology are described below. Recovery of electrolytes using the whole-body washdown procedures was measured during six mock trials using a 2-liter solution of artificial sweat. Recovery of Na^+^ and Cl^−^ was 102 to 103%, which suggests effective detection of electrolytes in the whole-body washdown collection system. The day-to-day CV for whole-body sweat [Na^+^] and [Cl^−^] in this study (trial 2b) was 8 to 10%.

### Trial design and protocols

Additional experimental procedures for each trial are described below. Table S2 provides a summary of descriptive data related to the exercise conditions, environment, and physiological outcome measures in each trial. Experimental procedures for the reference techniques used to measure regional and whole-body sweating rate and sweat [Cl^−^] have been described in more detail in previous publications (*11*, *12*).

#### Trial 1

Sweat was collected with the microfluidic and absorbent patches from 43 subjects (15 males and 28 females; 17 ± 1 year old; 64.3 ± 10.4 kg) from five sports (tennis, soccer, lacrosse, basketball, and track and field) during on-the-field/court, coach-led training sessions (22° to 34°C, 50 to 82% relative humidity). All sports were outdoors with the exception of basketball. Body mass was measured before and after exercise to the nearest 0.01 kg on a digital platform scale (Mettler Toledo ICS425s-BC300, Columbus, OH) while subjects wore minimal clothing (i.e., compression shorts/sports bra). Subjects were asked to towel dry before each body mass measurement. Subjects were allowed to consume water, a 6% carbohydrate-electrolyte solution, and sports nutrition products ad libitum during training. All drink bottles and nutrition products were massed before consumption, and the drink bottles and remaining food (or in many cases, the empty food wrapper) were massed after consumption (to the nearest 1 g; Ohaus CS2000, Pine Brook, NJ). When necessary, athlete’s urine loss during exercise was collected using a preweighed beaker/container and later massed (to the nearest 1 g; Ohaus CS2000, Pine Brook, NJ). Each participant’s energy expenditure during exercise was estimated using a Global Positioning System device (STATSports APEX Team Series, Newry, Ireland).

#### Trial 2a

Subjects cycled on an ergometer (Velotron SRAM, Pro, Chicago, IL) at moderate intensity [(159 ± 43 W; 62 ± 6% maximal oxygen uptake (VO_2max_); 82 ± 5% maximal heart rate (HR_max_)] for 90 min in a climate-controlled chamber (32°C, 25 to 50% relative humidity). Heart rate was monitored using telemetry (Polar Electro RS400; Lake Success, NY), and power output (watts) and cadence were recorded every 10 min. Energy expenditure (kilocalorie) was calculated from the cycling work rate (*36*). Subjects were provided a commercially available 6% carbohydrate-electrolyte solution to drink ad libitum during exercise. Immediately before and after exercise, nude body mass was recorded using a digital platform scale (KCC300 platform and IND439 reader; Mettler Toledo, Columbus, OH) to the nearest 0.01 kg. Subjects were asked to towel dry before each body mass measurement.

The whole-body washdown method was used to determine sweat [Na^+^] and sweat [Cl^−^] from the entire body (*16*, *37*). Before exercise, subjects’ whole bodies were rinsed with 5.0 liters of deionized water using a compression sprayer (model 010PEXG, Gilmour, Somerset, PA) and then dried with electrolyte-free paper towels (Wypall L-40, Kimberly-Clark, Irving, TX). Next, subjects donned compression shorts/sport bra and a heart rate monitor that had been previously rinsed with deionized water to remove any electrolytes and air-dried. Subjects did not wear socks or shoes during the trial. During exercise, care was taken to avoid sweat drippage. Two front (lower body, 2.9 to 3.1 m/s and upper body, 2.3 to 3.0 m/s) fans and one rear (1.5 to 2.0 m/s) fan were used to promote evaporative cooling. Subjects were given an electrolyte-free paper towel to absorb sweat from their face, neck, front torso, and arms. While double-gloved, study investigators wiped the subject’s back with an electrolyte-free paper towel to prevent dripping of sweat. The cycle ergometer seat and handlebars were covered with a plastic bag.

At the end of the 90 min of cycling exercise, the subjects stepped off the ergometer and directly into the washdown chamber that was positioned next to the cycle ergometer. The post-exercise washdown chamber consisted of a bale bag (Farm Bag Film Division, Glenford, OH) inside a steel frame (1.6 meters by 0.8 meters by 0.9 meters). The shorts/sport bra, heart rate monitor strap, and paper towels used to wipe the subjects’ sweat were hung to air dry. Next, the nude subject was rinsed thoroughly with deionized water (using a compression sprayer, N-80; Tabor Tools, Kibbutz Beit Rimon, Israel) to ensure removal of all sweat electrolytes from the skin and hair. Five liters of deionized water was prepared, of which a 200-ml sample was separated into aliquots for pre-rinse analysis, and the remaining 4.8 liters was used for rinsing the subject. After rinsing, the subject dried off with electrolyte-free paper towels and stepped out of the washdown chamber. The heart rate monitor and subject’s shorts/sport bra and all paper towels, gauze, elastic netting, Tegaderm part of the patches, and investigators’ outer gloves that touched the subject during exercise were put in the bottom of the bale bag (with the post-rinse deionized water). After the contents collected at the bottom of the bale bag were thoroughly mixed, a post-rinse sample was collected for electrolyte analysis (via ion chromatography; Dionex ICS-3000) (*37*). Whole-body sweat [Na^+^] and [Cl^−^] were determined from dilution calculations based on the measured [Na^+^] and [Cl^−^] in the post-rinse solution, the known volume of deionized water added to the bale bag (4.8 liters), and sweat loss.

#### Trial 2b

A subset of 12 subjects (8 males and 4 females) in trial 2a completed two additional trials (under standardized conditions) 2 to 8 days apart (at the same time of day) to determine the day-to-day reliability of regional sweating rate and sweat [Cl^−^] measured with the microfluidic patch and absorbent patch. To ensure consistency between trials, the subjects reported to the laboratory after abstaining from caffeine, alcohol, and vigorous exercise for 24 hours and food for 2 hours. In addition, subjects were asked to consume a consistent diet in the 48 hours preceding each trial and record all food and fluid intake in that time frame. Diets were analyzed using Nutribase Software (NB19Pro+, CyberSoft Inc.; Phoenix, AZ). Subjects were asked to drink 500 ml of water 2 hours before the trials. A urine sample was collected for the assessment of baseline urine specific gravity (USG; Atago Pen Refractometer, 3741-E03, Tokyo, Japan).

Subjects cycled on an ergometer (Velotron SRAM, Pro, Chicago, IL) at moderate intensity for 90 min in a climate-controlled chamber. Dietary analysis confirmed that energy (5404 ± 2576 versus 5166 ± 2303 kcal, *P* = 0.55), water (7.2 ± 4.1 versus 7.2 ± 3.5 liters, *P* = 0.99), and Na^+^ intake (7457 ± 2915 versus 7082 ± 2543 mg, *P* = 0.56) were consistent in the 48 hours leading up to each trial. There was no difference in baseline USG (1.013 ± 0.010 versus 1.010 ± 0.010, *P* = 0.21) or body mass (75.96 ± 13.68 versus 75.88 ± 13.76 kg, *P* = 0.77) between trials. All experimental procedures for measuring regional sweating rate, regional sweat [Cl^−^], and whole-body sweating rate were the same as in trial 2a. Fluid intake during exercise (0.891 ± 0.469 versus 0.890 ± 0.473 liters, *P* = 0.85) and net fluid balance (−1.18 ± 0.93 versus −1.21 ± 0.93%, *P* = 0.43) were consistent between trials. As expected, there were also no differences in absolute workload (160 ± 45 versus 162 ± 47 W, *P* = 0.21), relative intensity (63 ± 5 versus 64 ± 6% maximal oxygen uptake, *P* = 0.20; 82 ± 5 versus 82 ± 5% maximal heart rate, *P* = 0.38), or environmental conditions (32.0° ± 0.1° versus 31.9° ± 0.1°C, *P* = 0.24; 51 ± 1 versus 51 ± 1% relative humidity, *P* = 0.12) between trials.

#### Trial 3

Microfluidic regional sweating rate and whole-body sweating rate were measured in 312 subjects (194 males and 118 females) to develop a whole-body sweating rate prediction equation. All subjects in trial 1 (*n* = 43) and trial 2 (*n* = 45) were included in this dataset. Data were collected in the field (*n* = 198) and laboratory (*n* = 114) in a variety of athletes (43 to 150 kg, 15 to 45 years) and environmental conditions (21° to 35°C, 25 to 82% relative humidity, wind 0 to 7 m/s). In the field, energy expenditure was estimated using a Global Positioning System device (STATSports APEX Team Series). See tables S1 and S2 for more details. Experimental procedures in the field and laboratory were the same as described above for trials 1 and 2, respectively.

### Statistical analyses

Analyses were carried out using Statistical Analysis Software version 9.4 (SAS Institute, Cary, NC), Minitab 17 Statistical Software (Minitab, State College, PA), and JMP Pro version 15.1 (SAS Institute, Cary, NC). The significance level for all statistical tests was set at α = 0.05. Shapiro-Wilk tests were conducted to assess normality of the data, and Levene’s tests were used to assess homogeneity of variance. Data are shown as means ± SD. Paired *t* tests were used to determine mean differences between microfluidic and absorbent patch measures of regional sweating rate and sweat [Cl^−^]. To determine the intramethod test-retest reliability, paired-sample *t* tests and CVs were used.

Pearson product-moment correlations were conducted to determine the relations between the microfluidic patch and absorbent patch and between regional and whole-body measures of sweating rate and sweat [Cl^−^]. In instances of deviation from normality or homogeneity of variance, data were natural log-transformed to meet assumptions before analyses (Fig. 3B). Where natural log transformation did not resolve deviation from normal distribution, nonparametric Spearman correlation analysis was conducted (Fig. 4B).

Multiple regressions with diagnostic tests on residuals were used to develop prediction models for whole-body sweat [Cl^−^] and whole-body sweating rate. The prediction strength of regression models was assessed via coefficients of determination (*r*^2^) (*38*). Quantitative agreement between predicted and measured was assessed using the CCC, which measures the degree of departure between *x*-axis and *y*-axis values relative to perfect concordance, or the line of identity (*39*). A CCC > 0.80 is considered very good agreement (*40*). Prediction model error was quantified using mean absolute error, mean absolute percentage error, and RMSE.

## Supplementary Material

http://advances.sciencemag.org/cgi/content/full/6/50/eabe3929/DC1

Adobe PDF - abe3929_SM.pdf

Skin-interfaced microfluidic system with personalized sweating rate and sweat chloride analytics for sports science applications

## SUPPLEMENTARY MATERIALS

Supplementary material for this article is available at http://advances.sciencemag.org/cgi/content/full/6/50/eabe3929/DC1
