# Supplementary material for: Skin-interfaced microfluidic system with personalized sweating rate and sweat chloride analytics for sports science applications
Source: Sci Adv. 2020 Dec 11;6(50):eabe3929. doi: 10.1126/sciadv.abe3929 (PMC7732194; doi:10.1126/sciadv.abe3929)
Supplement: http://advances.sciencemag.org/cgi/content/full/6/50/eabe3929/DC1 [file supp_6_50_eabe3929__index.html]

Science Advances | Science AdvancesAAASSearchScience AdvancesMenu

## Supplementary Materials

# Skin-interfaced microfluidic system with personalized sweating rate and sweat chloride analytics for sports science applications

Lindsay B. Baker, Jeffrey B. Model, Kelly A. Barnes, Melissa L. Anderson, Stephen P. Lee, Khalil A. Lee, Shyretha D. Brown, Adam J. Reimel, Timothy J. Roberts, Ryan P. Nuccio, Justina L. Bonsignore, Corey T. Ungaro, James M. Carter, Weihua Li, Melissa S. Seib, Jonathan T. Reeder, Alexander J. Aranyosi, John A. Rogers, Roozbeh Ghaffari

Download Supplement

**This PDF file includes:**

- Figs. S1 to S4
- Tables S1 and S2

**Files in this Data Supplement:**

- Adobe PDF - abe3929\_SM.pdf
