# Supplementary material for: Skin-interfaced microfluidic system with personalized sweating rate and sweat chloride analytics for sports science applications
Source: Sci Adv. 2020 Dec 11;6(50):eabe3929. doi: 10.1126/sciadv.abe3929 (PMC7732194; doi:10.1126/sciadv.abe3929)
Supplement: http://advances.sciencemag.org/cgi/content/full/6/50/eabe3929/DC1 [file supp_6_50_eabe3929__1.pdf]

Science Advances
AAAS

advances.sciencemag.org/cgi/content/full/6/50/eabe3929/DC1

Supplementary Materials for

Skin-interfaced microfluidic system with personalized sweating rate and sweat chloride analytics for sports science applications

Lindsay B. Baker*, Jeffrey B. Model, Kelly A. Barnes, Melissa L. Anderson, Stephen P. Lee, Khalil A. Lee, Shyretha D. Brown, Adam J. Reimel, Timothy J. Roberts, Ryan P. Nuccio, Justina L. Bonsignore, Corey T. Ungaro, James M. Carter, Weihua Li, Melissa S. Seib, Jonathan T. Reeder, Alexander J. Aranyosi, John A. Rogers*, Roozbeh Ghaffari*

*Corresponding author. Email: lindsay.baker@pepsico.com (L.B.B.); jrogers@northwestern.edu (J.A.R.); rooz@northwestern.edu (R.G.)

Published 11 December 2020, *Sci. Adv.* **6**, eabe3929 (2020)
DOI: 10.1126/sciadv.abe3929

This PDF file includes:

Figs. S1 to S4
Tables S1 and S2

Supplementary Figure 1. CONSORT flow diagram for study enrollment, participant exclusion, and data exclusion for Trial 1.

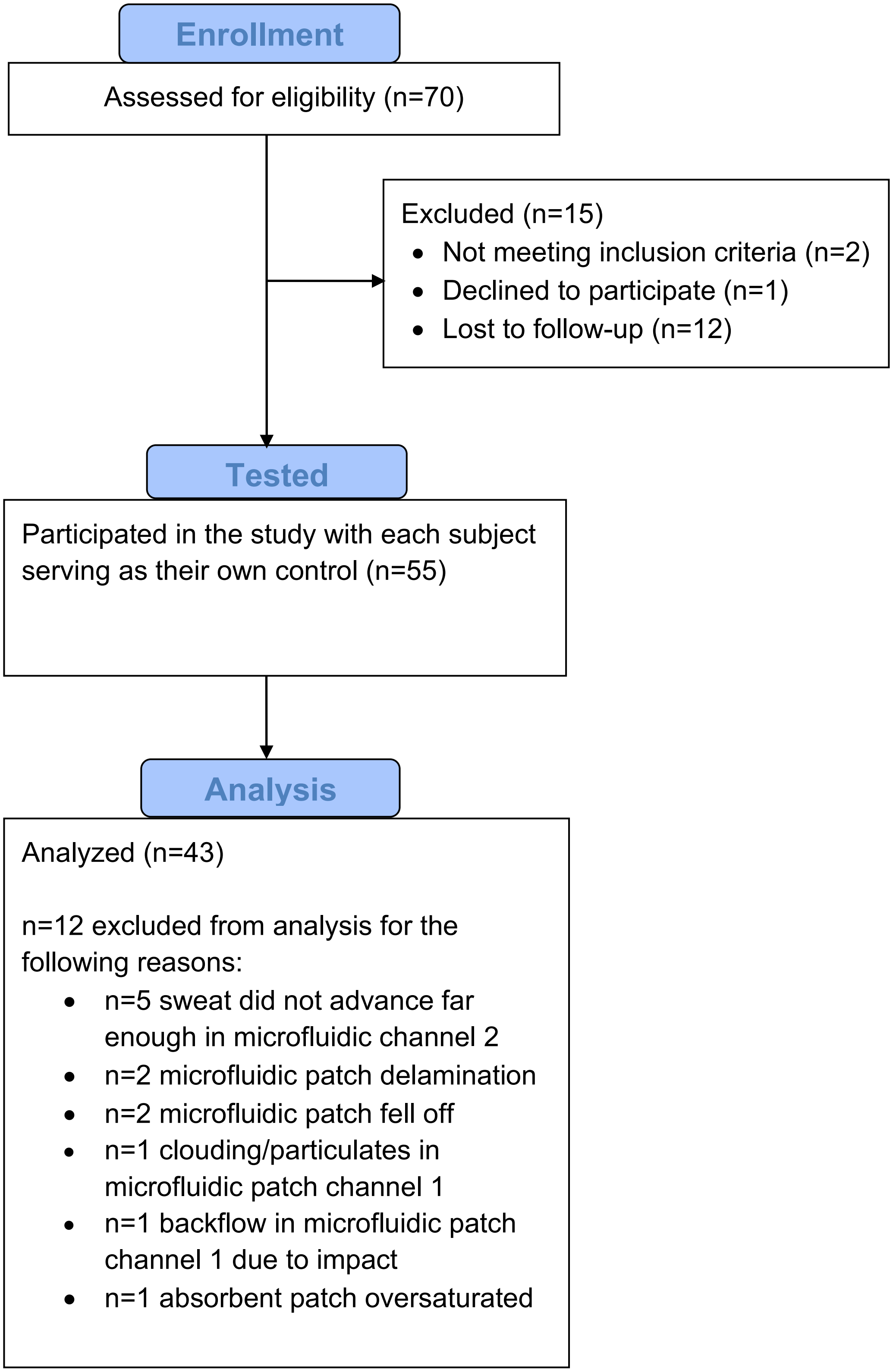

Supplementary Figure 2. CONSORT flow diagram for study enrollment, participant exclusion, and data exclusion for Trial 2.

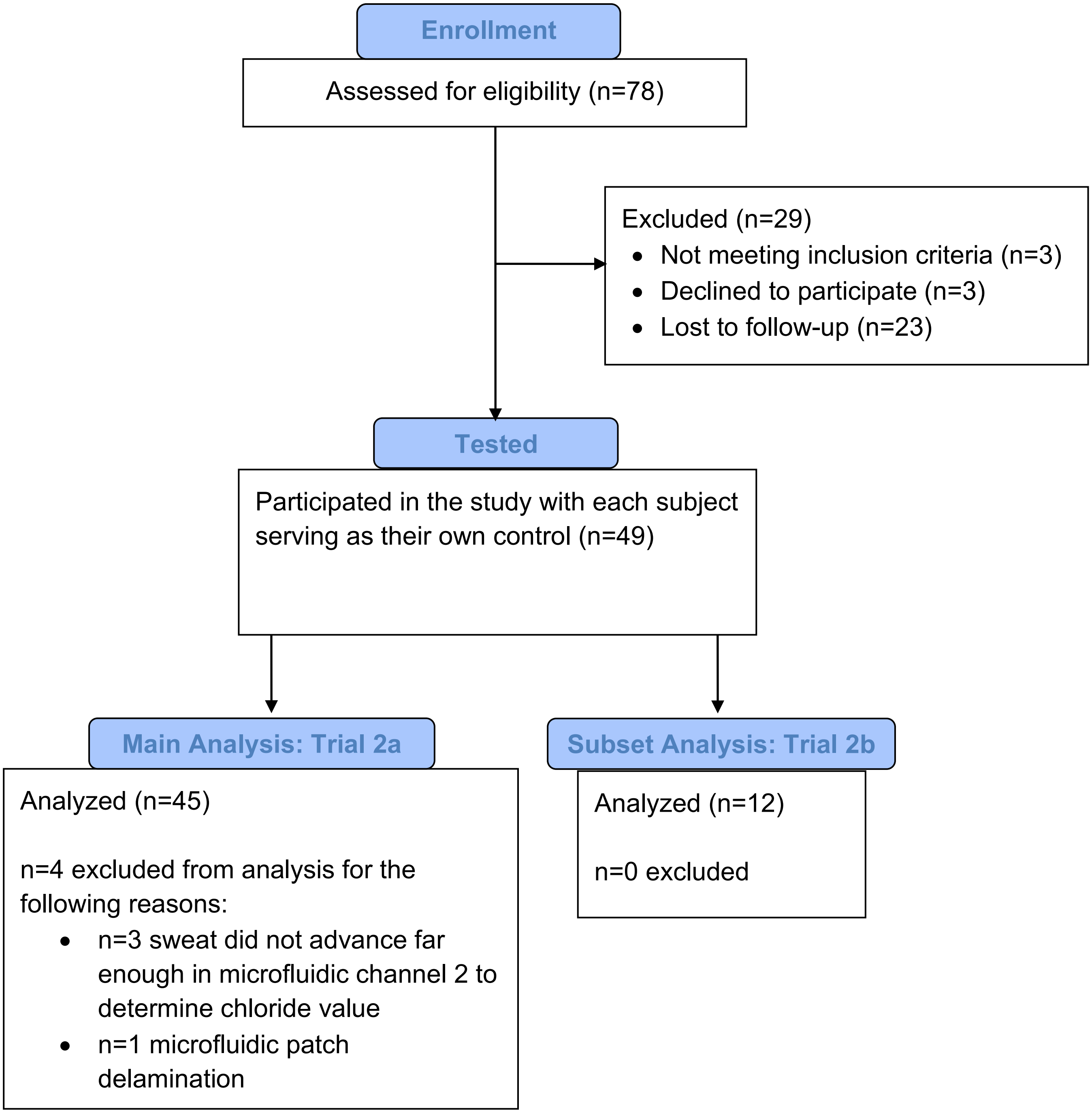

Supplementary Figure 3. CONSORT flow diagram for study enrollment, participant exclusion, and data exclusion for Trial 3.

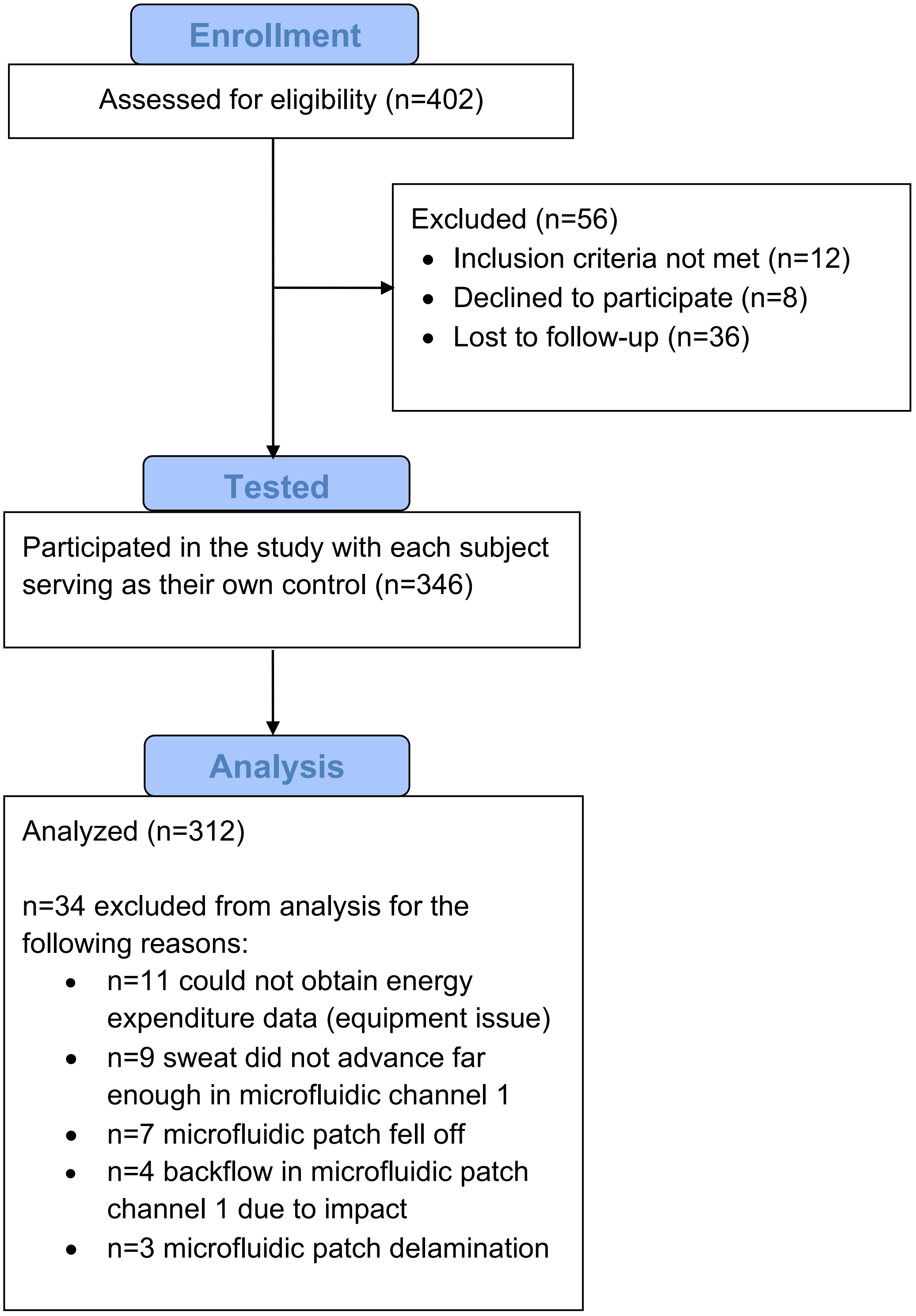

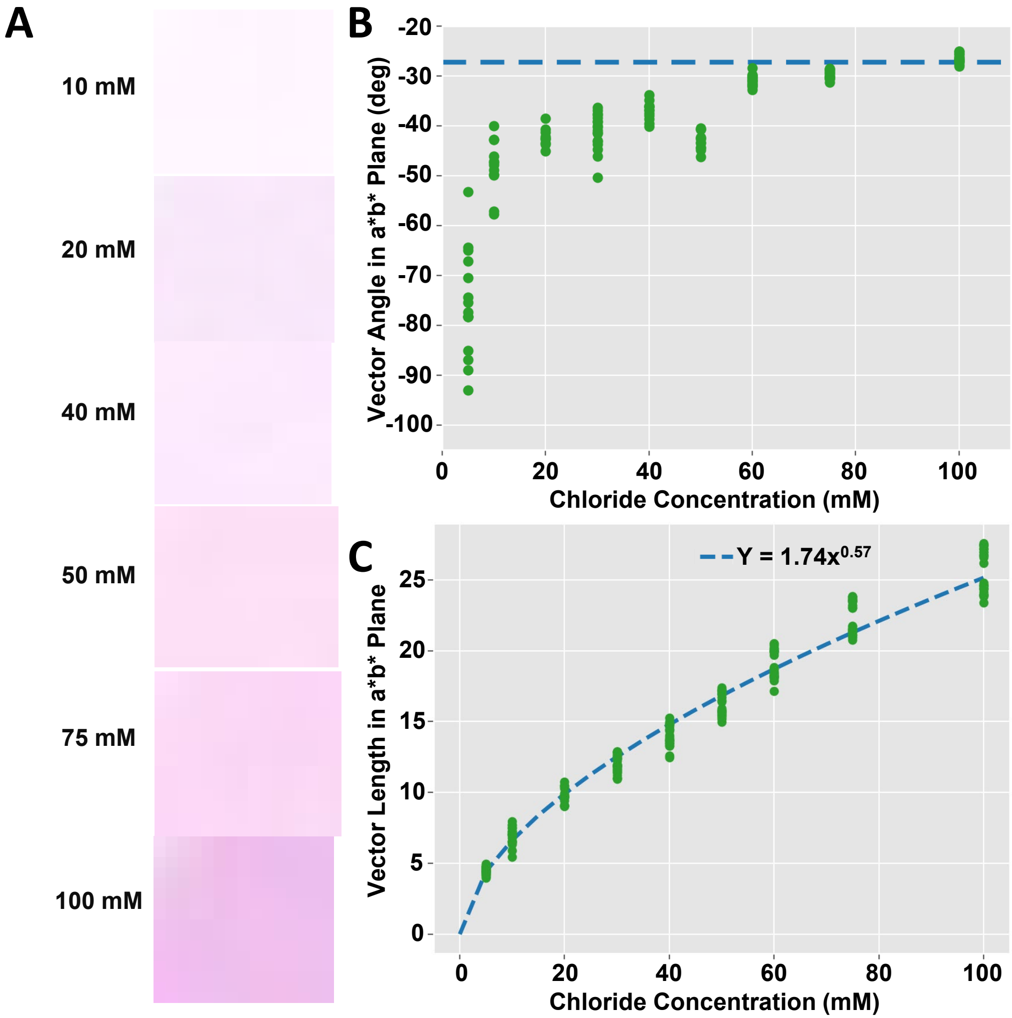

Supplementary Figure 4. Colorimetric analysis of chloride concentrations in microfluidic patch. (A) Photos of the chloride microchannel showing the increase in color intensity with concentration of perfused fluid. (B) Angle of the chloride microchannel color vector in the a*b* plane of CIELAB color space as a function of concentration. The angle asymptotically approaches -27.2 degrees. (C) Length of the chloride microchannel color vector projected onto -27.2 degrees as a function of chloride concentration. The corresponding power-law fit was used to measure chloride concentration from the colors measured from patch photos.

Supplementary Table 1. Subject characteristics for the athletes that participated in Trials 1-3.

	**Trial 1**	**Trial 2a**	**Trial 2b**	**Trial 3**
Objectives	Compare microfluidic patch and absorbent patch results for regional sweating rate and sweat [Cl^-]	Compare microfluidic patch and absorbent patch results for regional sweating rate and sweat [Cl^-]<br><br>Determine the relation between the microfluidic patch and whole body sweating rate and sweat [Cl^-]	Determine the day-to-day coefficient of variation of the microfluidic patch in measuring regional sweating rate and sweat [Cl^-]	Develop whole body sweating rate prediction equation
Location	Field	Laboratory	Laboratory	Field and Laboratory
Sample size: total (M/F)	n=43 (15 M, 28 F)	n=45 (31 M, 14 F)	Subset of Study 2a: N=12 (8 M, 4 F), tested 2x each	n=312 (194 M, 118 F)
Athlete type	Youth competitive individual sport and team sport athletes	Range of adult recreational exercisers to adult competitive endurance athletes	Range of adult recreational exercisers to adult competitive endurance athletes	Adult recreational exercisers; youth and adult competitive individual sport, team sport, and endurance athletes
VO_{2max} (ml/kg/min)	NA	48.6±8.3	48.2±7.0	NA
Ethnicity and Race	Non-Hispanic Caucasian (n=28), Non-Hispanic Asian (n=6), Hispanic Caucasian (n=6), Non-Hispanic African American (n=2), Hispanic American Indian/Alaska Native (n=1)	Non-Hispanic Caucasian (n=40), Hispanic Caucasian (n=2), Non-Hispanic Asian (n=2), Non-Hispanic African American (n=1)	Non-Hispanic Caucasian (n=11), Hispanic Caucasian (n=1)	Non-Hispanic Caucasian (n=204), Hispanic Caucasian (n=49), Non-Hispanic African American (n=29), Non-Hispanic Asian (n=21), Non-Hispanic Native Hawaiian/Pacific Islander (n=4), Hispanic American Indian/Alaska Native (n=1), Non-Hispanic Caucasian and African American (n=2), Non-Hispanic Caucasian and Asian (n=2)
Age (years)	17±1	34±4	34±4	23±9
Body Mass (kg)	64.29±10.38	75.29±12.50	75.96±13.68	70.47±15.08
Body Surface Area (m^2)	1.75±0.17	1.92±0.19	1.93±0.22	1.85±0.23

Values are mean±SD.

Supplementary Table 2. Exercise and environmental descriptive data for Trials 1-3.

	**Trial 1**	**Trial 2a**	**Trial 2b**	**Trial 3**
Objectives	Compare microfluidic patch and absorbent patch results for regional sweating rate and sweat [Cl^-]	Compare microfluidic patch and absorbent patch results for regional sweating rate and sweat [Cl^-]<br><br>Determine the relation between the microfluidic patch and whole body sweating rate and sweat [Cl^-]	Determine the day-to-day coefficient of variation of the microfluidic patch in measuring regional sweating rate and sweat [Cl^-]	Develop whole body sweating rate prediction equation
Location	Field	Laboratory	Laboratory	Field and Laboratory
Mode of Exercise	Track and field (n=3), Basketball (n=2), Soccer (n=19), Tennis (n=13), Lacrosse (n=6)	Cycling	Cycling	Field: Track and field (n=7), Cross-country running (n=9), Basketball (n=39), Soccer (n=95), Tennis (n=27), Lacrosse (n=12), and American football (n=9)<br><br>Laboratory: Cycling (n=101) and Running (n=13)
Exercise Duration (min)	87.5±25.9	90	90	83.4±24.6
Energy Expenditure (kcal)	313±125	961±221	Day 1: 970±239<br>Day 2: 976±248	506±279
Ambient Temperature (°C)	30.1±2.6	32.2±0.2	Day 1: 32.0±0.1<br>Day 2: 31.9±0.1	29.3±3.7
Relative Humidity (%)	65±11	39±12	Day 1: 51±1<br>Day 2: 51±1	58±13
Wind speed (m/s)	1.9±1.0	Front fans: lower body 2.9-3.1 m/s, upper body 2.3-3.0 m/s; Rear fan: 1.5-2.0 m/s	Day 1 and 2: Front fan on upper body: 3.0 m/s	1.8±1.2
WBGT (°C)	28.8±2.6	25.4±1.7	Day 1: 26.9±0.2<br>Day 2: 27.0±0.2	26.5±4.0
Whole Body Sweating Rate (L/h)	0.90±0.25	0.95±0.32	Day 1: 1.07±0.50<br>Day 2: 1.09±0.49	0.92±0.33
Sweat Loss (L)	1.32±0.60	1.43±0.48	Day 1: 1.61±0.76<br>Day 2: 1.64±0.73	1.25±0.79

Fluid Intake (L)	1.16±0.56	0.87±0.49	Day 1: 0.89±0.47<br>Day 2: 0.89±0.47	0.88±0.55
Fluid Balance (%)	-0.22±0.49	-0.98±0.73	Day 1: -1.18±0.93<br>Day 2: -1.21±0.93	-0.66±0.74

Values are mean±SD.
